# Towards building a trustworthy pipeline integrating Neuroscience Gateway and Open Science Chain

**DOI:** 10.1093/database/baae023

**Published:** 2024-04-03

**Authors:** S Sivagnanam, S Yeu, K Lin, S Sakai, F Garzon, K Yoshimoto, K Prantzalos, D P Upadhyaya, A Majumdar, S S Sahoo, W W Lytton

**Affiliations:** San Diego Supercomputer Center, University of California, San Diego, 9500 Gilman Drive, La Jolla, CA 92093, USA; Biomedical Engineering, SUNY Downstate Health Sciences University, 450 Clarkson Avenue, Brooklyn, NY 11203, USA; San Diego Supercomputer Center, University of California, San Diego, 9500 Gilman Drive, La Jolla, CA 92093, USA; San Diego Supercomputer Center, University of California, San Diego, 9500 Gilman Drive, La Jolla, CA 92093, USA; San Diego Supercomputer Center, University of California, San Diego, 9500 Gilman Drive, La Jolla, CA 92093, USA; San Diego Supercomputer Center, University of California, San Diego, 9500 Gilman Drive, La Jolla, CA 92093, USA; San Diego Supercomputer Center, University of California, San Diego, 9500 Gilman Drive, La Jolla, CA 92093, USA; School of Medicine, Case Western University, 9501 Euclid Ave, Cleveland, OH 44106, USA; School of Medicine, Case Western University, 9501 Euclid Ave, Cleveland, OH 44106, USA; San Diego Supercomputer Center, University of California, San Diego, 9500 Gilman Drive, La Jolla, CA 92093, USA; School of Medicine, Case Western University, 9501 Euclid Ave, Cleveland, OH 44106, USA; Biomedical Engineering, SUNY Downstate Health Sciences University, 450 Clarkson Avenue, Brooklyn, NY 11203, USA

## Abstract

When the scientific dataset evolves or is reused in workflows creating derived datasets, the integrity of the dataset with its metadata information, including provenance, needs to be securely preserved while providing assurances that they are not accidentally or maliciously altered during the process. Providing a secure method to efficiently share and verify the data as well as metadata is essential for the reuse of the scientific data. The National Science Foundation (NSF) funded Open Science Chain (OSC) utilizes consortium blockchain to provide a cyberinfrastructure solution to maintain integrity of the provenance metadata for published datasets and provides a way to perform independent verification of the dataset while promoting reuse and reproducibility. The NSF- and National Institutes of Health (NIH)-funded Neuroscience Gateway (NSG) provides a freely available web portal that allows neuroscience researchers to execute computational data analysis pipeline on high performance computing resources. Combined, the OSC and NSG platforms form an efficient, integrated framework to automatically and securely preserve and verify the integrity of the artifacts used in research workflows while using the NSG platform. This paper presents the results of the first study that integrates OSC–NSG frameworks to track the provenance of neurophysiological signal data analysis to study brain network dynamics using the Neuro-Integrative Connectivity tool, which is deployed in the NSG platform.

**Database URL**: https://www.opensciencechain.org.

## Introduction

Neuroscience research generates vast amounts of diverse experimental data, including imaging and neurophysiological signal data that forms the foundation for data-driven computational models aimed at unraveling the complexities of neuronal networks, including brain functions. Collaborative data reuse (1–4) within the field fosters the development and sharing of critical workflow artifacts, including data preprocessing scripts, analysis pipelines and code libraries. These artifacts, accumulating over time, become valuable resources for the broader neuroscience community, facilitating efficient and transparent research endeavors.

While sharing data and metadata information, the trustworthiness of the shared information is pivotal in determining the reliability of the data in subsequent studies. Data provenance is a vital component in the sharing and reuse of scientific data (5) and it can be crucial in deciding whether the data can be trusted. Metadata elements needed to ensure data trustworthiness, such as provenance information describing who, what, why, which, when and how the data were generated and analyzed, must be maintained for the consistent reuse of data. An additional important facet of maintaining integrity is to ensure that the data or its associated metadata have not been altered, either unintentionally or maliciously. When the scientific dataset evolves or is reused in workflows creating derived datasets, the integrity of the dataset and its metadata, including provenance, needs to be securely preserved while providing assurances that they are not altered from their original version.

A novel approach to ensure the integrity of data and metadata is to safeguard their integrity by utilizing a distributed ledger system, specifically blockchain technology (6). The blockchain allows transactions (e.g. metadata or hash of data) to be securely stored and verified without a central authority. Blockchain’s ‘append-only’ structure prevents altering or deleting previously entered data. Therefore, data in the blockchain ledger are verifiable, timestamped and immutable, which is essential for reproducibility and audits.

We present findings from the development of an integrated framework that incorporates metadata and integrity information from neuroscience experiments conducted on the Neuroscience Gateway (NSG), a freely available resource via which multiple neuroscience data processing and modeling software are made available on high-performance computing resources (HPC), into the Open Science Chain (OSC), a free consortium blockchain-based platform. This integration aims to fortify the trustworthiness of data used in neuroscience workflows, ultimately promoting data reuse, and advancing collaborative research efforts in the field.

## Existing frameworks

### Neuroscience Gateway

The NSF- and NIH-funded NSG has been in operation since 2013 (7–11). It serves the neuroscience community by providing easy access to large number of software and pipelines running primarily on HPC resources available at academic supercomputer centers across the USA. NSG is open to neuroscience researchers from academic institutions, research institutes and non-profit organizations located in the USA and other countries and currently supports over 1600 registered researchers from the USA and other international countries. NSG provides about 20 computational neuroscience and data processing tools, software and pipelines that are optimally installed on various supercomputers. For example, NSG software applications include NEURON (12) for building and using computational models of neurons and networks of neurons, EEGLAB (13) for processing data from electroencephalography, magnetoencephalography and other electrophysiological signals, NEST (14) for creating spiking neural network models and the Neuro-Integrative Connectivity (NIC) (15) for performing brain functional connectivity network analysis. Researchers primarily interact with NSG through an easy-to-use web front-end interface allowing flexible use of the many neuroscience software tools and pipelines on various supercomputers. NSG’s technologically streamlined front-end web interface allows users to easily upload computational models or input data, specify parameters related to neuroscience tools and HPC resources, query running job status, receive job completion notices and retrieve output data. Each application possesses its own distinct set of metadata requirements tailored to the specific requirements of the research or analysis at hand. These unique metadata parameters are integral in ensuring the reproducibility of experiments and analyses. NSG currently does not have controls in place to collect and store metadata associated with each execution of the application to encourage reuse and reproducibility as part of the Findable Accessible Interoperable Reusable (FAIR) guidelines (16).

### Open Science Chain

The NSF-funded OSC (17–19) utilizes consortium blockchain to provide a cyberinfrastructure solution to maintain integrity and provenance associated with published datasets and provides a way to perform independent verification of the dataset while promoting reuse and reproducibility. The OSC, implemented using the open-source Hyperledger Fabric Framework (20), allows researchers or projects to store the cryptographic hash of the data (e.g. SHA256 checksum) as a manifest in the blockchain along with the metadata. The actual data are stored off-chain as storing large amounts of data in the blockchain is inefficient, especially since some scientific datasets tend to be in the multi-terabyte range or larger in size. OSC is agnostic to the type of data whose verification information is stored in the blockchain. OSC will generate an identifier for the information stored on the blockchain that uniquely ties together metadata elements such as contributor information, location of the data and cryptographic hash of the data. Blockchain’s ‘append only’ structure prevents altering or deleting previously entered data, and the distributed, replicated nature of the blockchain ledger assures that multiple copies exist. Data in the blockchain ledger are therefore verifiable and immutable, which is essential for reproducibility and audits. OSC provides programmatic access to the blockchain through a python-based command line utility (21) and allows registered external platforms and hubs to connect and use the blockchain.

## Implementation

We stored the standardized metadata information associated with the data generated by the NIC tool (15, 22, 23), which is one of the software tools available within NSG framework, into the OSC to study the reuse and reproducibility of the application. This work leverages the existing OSC command line utility to store and update the metadata and any workflows utilizing the metadata in the blockchain. The integration of this NSG-provided NIC software with OSC is essential for developing and testing the prototype. This will allow us to extend and generalize this to other NSG-provided software as we have envisioned as the overall integration between NSG and the OSC framework.

### Metadata of NIC on NSG

The NIC tool is a compositional workflow-based tool that analyzes brain functional connectivity patterns in neurological disorders using electrophysiological signal data such as electroencephalogram (EEG) recordings. The NIC tool uses a modular software architecture to support end-to-end EEG data analysis, including the use of JavaScript Object Notation (JSON)-based data format for efficient analysis, computation of coupling measures representing interactions between brain regions, and the use of algebraic topology methods to characterize brain interaction patterns. The various modules of the NIC pipeline are described below:

#### NIC data pre-processing module

The NIC data pre-processing module transforms EEG data storing using the widely used European Data Format Plus Annotations specifications, which are inefficient for use in brain network analysis, into an optimized structure called Cloud wave Signal Format (CSF) (22). The CSF files store channel-wise EEG recordings in a channel-specific format spanning multi-second time windows and incorporate semantic annotations mapped to terms defined in an epilepsy domain ontology (24). TheJSON-based CSF file format is self-descriptive, containing study metadata, channel-specific metadata, clinical event annotations and fragments of signal data. The CSF format enhances human readability, facilitates the storage of EEG data within a distributed storage system, supports efficient random access and offers flexibility for integration with parallel processing methods.

#### NIC signal coupling measure computation module

This module computes quantitative measures of the coupling between signal recordings from different electrodes. The module computes several coupling measures, including the non-linear correlation coefficient developed by Pijn *et al*. (25), phase coherence developed by Mormann *et al*. (26) and Pearson’s linear correlation coefficient (27). This module ingests signal data stored in CSF files, ideally generated with the companion NIC data pre-processing module and generates the relevant coupling measure values, which are stored in text files for subsequent analysis.

#### NIC topological data analysis module

The topological data analysis (TDA) module uses coupling measure values to generate algebraic topology structures such as simplicial complexes representing high-dimensional interaction patterns (28) using persistent homology methods that have been implemented in open-source libraries such as the GUDHI library (29) to generate these algebraic topology structures. The NIC TDA module generates a single output file with values corresponding to the birth, death and dimension values of algebraic topology structures called homology classes (30). These algebraic topology values are subsequently analyzed using statistical methods and machine learning algorithms.

Each module of the NIC workflow tool involves the recording of unique provenance metadata elements to accurately record the context of each experimental study to support subsequent reproducibility and enable compliance with FAIR guidelines for neurological studies in the future. We note that additional features need to be implemented in the current version of the NIC workflow tool to meet the FAIR guidelines using the S3 framework, which consists of metadata describing Study Method, Study Data and Study Instrument, as reference for neuroscience (31, 32).

**Figure 1.**
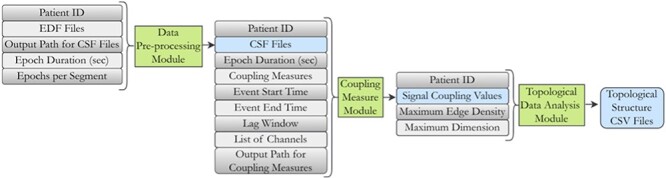
Each NIC module (green) has its own distinct set of metadata requirements to generate output.

Figure 1 illustrates the various metadata elements associated with each NIC module. Patient ID, Epoch duration, Epochs per segment, Event start and end times along with list of channels and maximum edge density and dimension are the various metadata elements involved in running the NIC pipeline. Capturing metadata related to the execution of the NIC pipeline, along with details about running the pipeline on computational resources, facilitates the reproducibility of results on the NSG. Additionally, this practice promotes reusability, as it enables fellow researchers to validate results by running the pipeline on comparable computational resources. The use of blockchain technology instills confidence in the authenticity of the recorded information, mitigating potential trust deficits. This secure and transparent ledger promotes accountability and fosters trust among researchers, facilitating the reproducibility of results on the NSG and encouraging the reusability of the pipeline on comparable computational resources.

#### Metadata related to using NSG

The NSG runs various computational neuroscience applications on supercomputers. To initiate running on NSG, researchers must select the neuroscience application and associated parameters to execute the application on the supercomputer. Information related to running on supercomputers includes number of servers, number of processors, estimated time to run on the supercomputers and name of the input file. Parameters specific to the application vary based on the application. The integration with OSC in the NSG deployed version of the NIC tool preserves the provenance and integrity information related to the data and metadata elements generated and used by the NIC modules as well as metadata that is associated with running the NIC tool through NSG on supercomputers.

### Development of modules for metadata capture from NSG

NSG effectively manages and archives all pertinent data associated with the execution of software applications on remote HPC resources by storing this information within a MySQL database. NSG incorporates a user mapping system, enabling the monitoring of individual usage of computational resources.

To facilitate the initial phase of integrating NSG with OSC, a new table was introduced within NSG’s MySQL database. This table serves the purpose of capturing essential data elements, including the job identifier (job id) for the neuroscience application, the status of job execution (such as ‘completed’, ‘queued’ or ‘running’) within the NIC module and the unique OSC-ID. This OSC-ID will be generated and logged upon the successful addition of the corresponding information associated with running a neuroscience application within NSG framework to the OSC blockchain.

### Integration with OSC

A programmatic module was developed to facilitate the transfer of relevant metadata as an artifact to the OSC blockchain. When a user executes NIC pipeline on NSG, the information related to that job, the NIC job id and the job status are stored in the newly created table of NSG. The job status is updated by daemons running as a part of the NSG code. Automated scripts were developed to invoke the submission process to OSC using the OSC command line utility. When the job status becomes completed, the metadata from the job gets extracted. This includes various metadata elements associated with each NIC module as well as information related to running the job on the supercomputer. The cryptographic checksum of the data used in the experiment was calculated and appended to this artifact. The information is added in a format that is supported by the OSC command line utility application programming interface (API). The OSC command line utility initiates connection to OSC and transfers the information to be stored in the blockchain. The chain code of the blockchain can identify duplicate entries in which case the transaction is rejected and the status is updated in NSG. Upon successful inclusion in the blockchain, an OSC-ID is generated and sent back to NSG which is then added to the MySQL table. When the data or metadata evolve for the same experiment, updates are identified and sent to the blockchain, thus maintaining the provenance of the data or metadata of the experiment. Scripts to verify the contributed information (at a regular interval) are managed through cron jobs.

### Results from prototype implementation using the NIC tool

**Figure 2.**
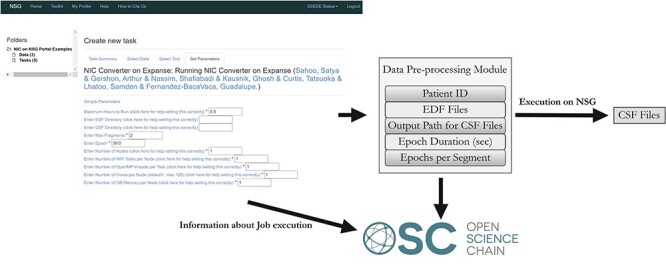
NIC module execution on NSG and passing on the metadata to OSC.

**Figure 3.**
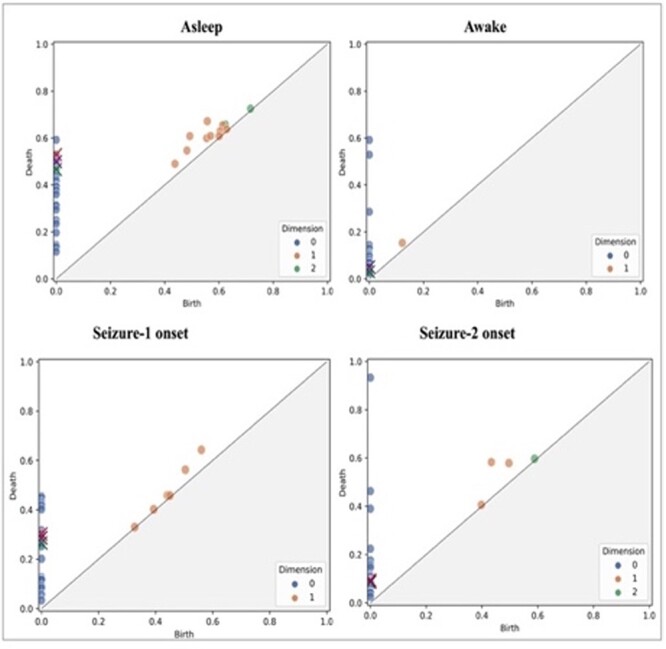
Scatter plots of homology classes for a patient (persistence diagrams) corresponding to asleep, awake, seizure one onset and seizure two onset states.

Figure 2 illustrates the execution of the NIC data pre-processing module on the NSG website, utilizing the San Diego Supercomputer Center’s Expanse HPC infrastructure. Once the execution of the NIC data pre-processing module is successfully completed on the HPC resource, an array of essential metadata pertaining to this process is extracted and sent to the OSC blockchain. This metadata encompasses critical details such as the duration of each EEG recording called epoch in seconds (Epoch Duration) and the number of epochs contained within each segment (Epochs per segment). As an example, Figure 3 is a visualization of homology classes generated by the NIC TDA module using results from the NIC signal coupling measure computation module. The lifespan and dimension information of these homology classes are used for binary classification of EEG recordings into seizure and non-seizure phases [our recent work provides additional details in this regard (33)]. To reproduce these results generated by the NIC tool, users need access to provenance metadata describing the specific coupling measure used in the NIC module (e.g. phase coherence measure) and the algebraic topology method used to generate the homology classes (e.g. persistent homology).

**Figure 4.**
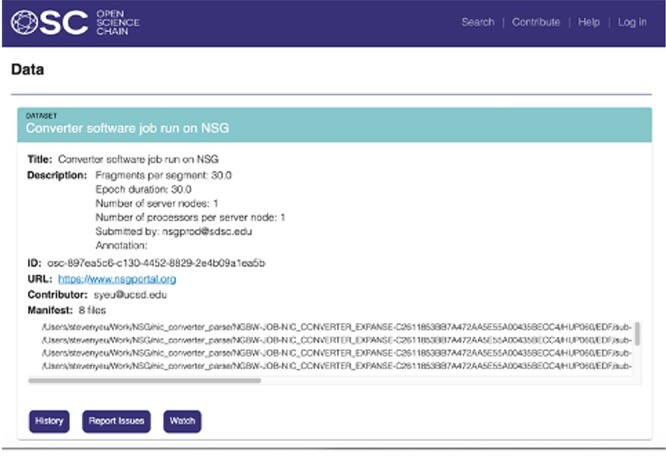
Data and metadata stored in the blockchain.

Additional provenance metadata information includes specific file path where the associated files are located, the cryptographic hash integrity information of the input and output files, the contributor email, information related to running the job on the HPC resource such as the number of processors used and the version of the NIC software is also included in the transmission. The information stored in the blockchain is shown in Figure 4. OSC provides flexibility to accommodate customized metadata fields. This capability enables projects or applications to tailor the metadata to their specific needs, enhancing the comprehensiveness and relevance of the information associated with their scientific endeavors. Using OSC’s search API, information already stored in the blockchain can be extracted for independent verification and future reuse. In the future, we plan to integrate the search API with the NSG portal which will allow researchers to search for datasets or workflow processes that have been run on NSG for reuse.

## Conclusion

We describe the results of our work in integrating NSG and OSC platforms to support reproducibility in neuroscience research using novel blockchain techniques with the NIC workflow tool serving as a representative case study. The upcoming phases of this project involve broadening the scope of integration to encompass additional applications within NSG framework, notably NEURON to facilitate integration of provenance metadata from other NSG applications. NEURON is a simulation framework tool frequently employed in computational neuroscience research for modeling individual and networks of neurons and its integration promises to further enhance the collaborative potential of NSG and OSC. In addition, we plan to explore the possibility of using the information stored in the blockchain to identify analogous files used in creating neuronal models or in EEG studies. This will help streamline the identification and utilization of relevant data in similar research. The effort will also explore integration of citation data that can help researchers track and reference prior work more efficiently, fostering a culture of knowledge sharing and intellectual collaboration. Additional science gateways, such as CIPRES Phylogenetics gateway (34) and COSMIC Cryo-EM gateway (35), utilize the identical gateway backend framework as NSG. Therefore, as OSC becomes integrated into NSG, seamless integration with these alternative gateways becomes feasible.

## Data Availability

All data provided in the aforementioned framework are publically available.
